# 3,3-Dinitro­azetidinium 2-hy­droxy­benzoate

**DOI:** 10.1107/S1600536810043825

**Published:** 2010-10-31

**Authors:** Rong Gao, Biao Yan, Tao Mai, Ying Hu, Yu-Lei Guan

**Affiliations:** a Department of Chemistry, School of Pharmacy, Fourth Military Medical University, Xi’an 710032 Shaanxi, People’s Republic of China; b School of Chemistry and Chemical Engineering, Yulin University, Yulin 719000 Shaanxi, People’s Republic of China; c School of Chemical Engineering, Northwest University, Xi’an 710069 Shaanxi, People’s Republic of China

## Abstract

In the title *gem*-dinitro­azetidinium 2-hy­droxy­benzoate salt, C_3_H_6_N_3_O_4_^+^·C_7_H_5_O_3_^−^, the azetidine ring is virtually planar, with a mean deviation from the plane of 0.0242 Å. The dihedral angle between the two nitro groups is 87.5 (1)°.

## Related literature

For related literature on 1,3,3-trinitro­azetidine and compounds prepared from its derivative 3,3-dinitro­azetidine, see: Archibald *et al.* (1990 ▶); Gao *et al.* (2009 ▶); Hiskey *et al.* (1992 ▶); Ma, Yan, Li, Guan *et al.* (2009 ▶); Ma, Yan, Li, Song & Hu (2009 ▶); Ma, Yan, Song *et al.* (2009 ▶); Ma *et al.* (2010 ▶); Yan *et al.* (2009 ▶, 2010 ▶).
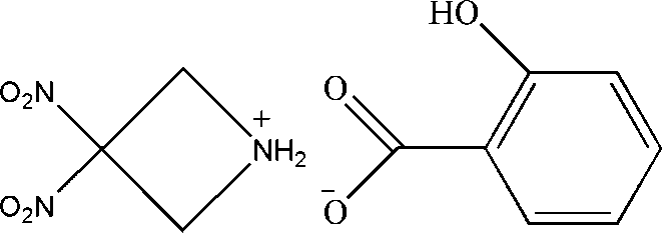


## Experimental

### 

#### Crystal data

C_3_H_6_N_3_O_4_^+^·C_7_H_5_O_3_^−^
*M* *_r_* = 285.22
Monoclinic, 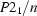

*a* = 11.174 (3) Å
*b* = 7.013 (2) Å
*c* = 16.661 (5) Å
β = 105.960 (5)°
*V* = 1255.3 (6) Å^3^
*Z* = 4
Mo *K*α radiation
μ = 0.13 mm^−1^
*T* = 296 K
0.36 × 0.26 × 0.19 mm

#### Data collection

Bruker APEX CCD area-detector diffractometer
Absorption correction: multi-scan (*SADABS*; Sheldrick, 2000 ▶) *T*_min_ = 0.955, *T*_max_ = 0.976
6012 measured reflections
2222 independent reflections
1504 reflections with *I* > 2σ(*I*)
*R*_int_ = 0.027

#### Refinement

*R*[*F* ^2^ > 2σ(*F* ^2^)] = 0.040
*wR*(*F* ^2^) = 0.128
*S* = 0.99
2222 reflections
183 parameters
H-atom parameters constrained
Δρ_max_ = 0.34 e Å^−3^
Δρ_min_ = −0.17 e Å^−3^

### 

Data collection: *SMART* (Bruker, 2003 ▶); cell refinement: *SAINT* (Bruker, 2003 ▶); data reduction: *SAINT*; program(s) used to solve structure: *SHELXS97* (Sheldrick, 2008 ▶); program(s) used to refine structure: *SHELXL97* (Sheldrick, 2008 ▶); molecular graphics: *SHELXTL* (Sheldrick, 2008 ▶); software used to prepare material for publication: *SHELXTL*.

## Supplementary Material

Crystal structure: contains datablocks I, global. DOI: 10.1107/S1600536810043825/ng5053sup1.cif

Structure factors: contains datablocks I. DOI: 10.1107/S1600536810043825/ng5053Isup2.hkl

Additional supplementary materials:  crystallographic information; 3D view; checkCIF report

## Figures and Tables

**Table 1:** Hydrogen-bond geometry (Å, °)

*D*—H⋯*A*	*D*—H	H⋯*A*	*D*⋯*A*	*D*—H⋯*A*
N1—H1*C*⋯O6	0.90	2.38	2.922 (2)	118
N1—H1*C*⋯O7	0.90	1.81	2.708 (2)	179
N1—H1*D*⋯O7^i^	0.90	1.96	2.720 (2)	141

Symmetry code: (i) 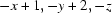.

**Fig. 1.**
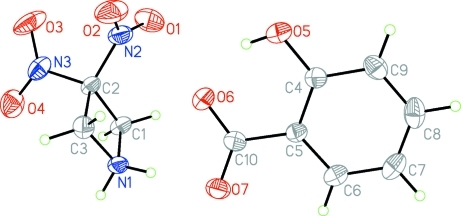
The molecular structure of (I), showing the atom-numbering scheme. Displacement ellipsoids are drawn at the 30% probability level and H atoms are drawn as spheres of arbitrary radius.

## supplementary crystallographic information

### Comment

Dinitro- and trinitro-derivatives of azetidine are of interest because they contain strained ring systems. This makes them good candidates for energetic materials (propellants or explosives). Azetidine-based explosives, such as 1,3,3-trinitroazetidine (TNAZ) (Archibald *et al.*, 1990) demonstrate excellent performance partly because of the high strain associated with the four-membered ring. As one of the important derivates of TNAZ, 3,3-dinitroazetidine (DNAZ) (Hiskey *et al.*, 1992;)can prepare a variety of solid energetic materials with high oxygen-balance (Ma, Yan, Li, Guan *et al.*, 2009; Gao *et al.*, 2009; Ma, Yan, Li, Song & Hu, 2009; Ma, Yan, Song *et al.*, 2009; Yan *et al.*, 2009; Yan *et al.*, 2010; Ma *et al.*, 2010). This paper reports synthesis and crystal structure of the title DNAZ salt.

### Experimental

A solution of DNAZ (0.30 g, 2.04 mmol), salicylic acid (0.28 ml, 2.04 mmol) in trichloromethane (15.0 ml) was stirred for 2 h. The reaction mixture was concentrated *in vacuo*, then a white solid began to precipitate. The solid product was washed with ethanol and purified by recrystallization from trichloromethane to give the pure colorless compound in 90.5% yield. The title compound (43 mg,0.15 mmol) was dissolved in chloroform (15 ml). Colorless crystals were isolated after several days. Elemental analysis calculated for C_10_H_11_N_3_O_7_: C 42.11, N 14.73, H 3.89%; found: C 47.44, N 14.80, H 3.89%. IR (KBr, cm^-1^): 3060.25, 1647.33, 1579.29, 1298.39, 1485.78, 1454.23, 706.64.

### Refinement

H atoms were placed at calculated idealized positions and refined using a riding model, with C—H distances in the range 0.93–0.97 Å, N—H distances 0.90 Å and O—H distances 0.82 Å.

### Crystal data

**Table:**

C_3_H_6_N_3_O_4_^+^·C_7_H_5_O_3_^−^	*F*(000) = 592
*M**_r_* = 285.22	*D*_x_ = 1.509 Mg m^−^^3^
Monoclinic, *P*2_1_/*n*	Melting point: 379.4 K
Hall symbol: -P2yn	Mo *K*α radiation, λ = 0.71073 Å
*a* = 11.174 (3) Å	Cell parameters from 1275 reflections
*b* = 7.013 (2) Å	θ = 2.5–21.9°
*c* = 16.661 (5) Å	µ = 0.13 mm^−^^1^
β = 105.960 (5)°	*T* = 296 K
*V* = 1255.3 (6) Å^3^	Block, colorless
*Z* = 4	0.36 × 0.26 × 0.19 mm

### Data collection

**Table:**

Bruker APEX CCD area-detector diffractometer	2222 independent reflections
Radiation source: fine-focus sealed tube	1504 reflections with *I* > 2σ(*I*)
graphite	*R*_int_ = 0.027
phi and ω scans	θ_max_ = 25.1°, θ_min_ = 2.0°
Absorption correction: multi-scan (*SADABS*; Sheldrick, 2000)	*h* = −12→13
*T*_min_ = 0.955, *T*_max_ = 0.976	*k* = −8→8
6012 measured reflections	*l* = −18→19

### Refinement

**Table:**

Refinement on *F*^2^	Secondary atom site location: difference Fourier map
Least-squares matrix: full	Hydrogen site location: inferred from neighbouring sites
*R*[*F*^2^ > 2σ(*F*^2^)] = 0.040	H-atom parameters constrained
*wR*(*F*^2^) = 0.128	*w* = 1/[σ^2^(*F*_o_^2^) + (0.0797*P*)^2^] where *P* = (*F*_o_^2^ + 2*F*_c_^2^)/3
*S* = 0.99	(Δ/σ)_max_ = 0.002
2222 reflections	Δρ_max_ = 0.34 e Å^−^^3^
183 parameters	Δρ_min_ = −0.17 e Å^−^^3^
0 restraints	Extinction correction: *SHELXL97* (Sheldrick, 2008), Fc^*^=kFc[1+0.001xFc^2^λ^3^/sin(2θ)]^-1/4^
Primary atom site location: structure-invariant direct methods	Extinction coefficient: 0.008 (2)

### Special details

**Table:**

Geometry. All e.s.d.'s (except the e.s.d. in the dihedral angle between two l.s. planes) are estimated using the full covariance matrix. The cell e.s.d.'s are taken into account individually in the estimation of e.s.d.'s in distances, angles and torsion angles; correlations between e.s.d.'s in cell parameters are only used when they are defined by crystal symmetry. An approximate (isotropic) treatment of cell e.s.d.'s is used for estimating e.s.d.'s involving l.s. planes.
Refinement. Refinement of *F*^2^ against ALL reflections. The weighted *R*-factor *wR* and goodness of fit *S* are based on *F*^2^, conventional *R*-factors *R* are based on *F*, with *F* set to zero for negative *F*^2^. The threshold expression of *F*^2^ > σ(*F*^2^) is used only for calculating *R*-factors(gt) *etc*. and is not relevant to the choice of reflections for refinement. *R*-factors based on *F*^2^ are statistically about twice as large as those based on *F*, and *R*- factors based on ALL data will be even larger.

### Fractional atomic coordinates and isotropic or equivalent isotropic displacement parameters (Å^2^)

**Table:**

	*x*	*y*	*z*	*U*_iso_*/*U*_eq_	
O7	0.58576 (13)	0.7941 (2)	0.06388 (8)	0.0563 (4)	
O6	0.46538 (12)	0.5959 (2)	0.10843 (9)	0.0629 (4)	
O5	0.53637 (13)	0.2679 (2)	0.15624 (10)	0.0619 (4)	
H5	0.4878	0.3566	0.1405	0.093*	
C4	0.67334 (16)	0.4982 (3)	0.12215 (10)	0.0380 (5)	
N1	0.36966 (14)	0.9772 (2)	0.05657 (9)	0.0447 (4)	
H1D	0.3660	1.0893	0.0297	0.054*	
H1C	0.4417	0.9169	0.0593	0.054*	
C5	0.65122 (17)	0.3202 (3)	0.15274 (11)	0.0407 (5)	
N3	0.10748 (16)	0.9419 (3)	0.10825 (12)	0.0580 (5)	
C10	0.56810 (18)	0.6383 (3)	0.09534 (11)	0.0439 (5)	
C3	0.34209 (17)	0.9937 (3)	0.13919 (11)	0.0471 (5)	
H3B	0.3181	1.1211	0.1514	0.056*	
H3A	0.4075	0.9437	0.1854	0.056*	
C2	0.23289 (16)	0.8586 (3)	0.10855 (11)	0.0412 (5)	
C6	0.7486 (2)	0.1923 (3)	0.18100 (11)	0.0511 (5)	
H6	0.7340	0.0722	0.2000	0.061*	
C1	0.25748 (18)	0.8552 (3)	0.02338 (11)	0.0479 (5)	
H1A	0.2765	0.7293	0.0061	0.057*	
H1B	0.1928	0.9160	−0.0201	0.057*	
C9	0.79343 (18)	0.5449 (3)	0.12150 (12)	0.0501 (5)	
H9	0.8089	0.6630	0.1010	0.060*	
N2	0.24179 (17)	0.6717 (3)	0.15310 (13)	0.0585 (5)	
O4	0.08623 (14)	1.0972 (3)	0.07553 (11)	0.0771 (5)	
C7	0.8681 (2)	0.2457 (4)	0.18067 (12)	0.0595 (6)	
H7	0.9340	0.1620	0.2011	0.071*	
O3	0.03912 (17)	0.8537 (3)	0.13847 (15)	0.0995 (7)	
C8	0.89002 (19)	0.4198 (4)	0.15066 (13)	0.0594 (6)	
H8	0.9703	0.4534	0.1500	0.071*	
O1	0.19839 (17)	0.5345 (3)	0.11130 (12)	0.0861 (6)	
O2	0.29006 (19)	0.6747 (3)	0.22818 (11)	0.0891 (6)	

### Atomic displacement parameters (Å^2^)

**Table:**

	*U*^11^	*U*^22^	*U*^33^	*U*^12^	*U*^13^	*U*^23^
O7	0.0650 (10)	0.0485 (9)	0.0622 (9)	0.0038 (7)	0.0288 (7)	0.0121 (7)
O6	0.0415 (8)	0.0663 (11)	0.0854 (11)	0.0040 (7)	0.0248 (7)	0.0167 (8)
O5	0.0492 (9)	0.0573 (10)	0.0826 (11)	−0.0109 (7)	0.0237 (8)	0.0107 (8)
C4	0.0390 (10)	0.0420 (11)	0.0340 (9)	−0.0047 (8)	0.0115 (7)	−0.0025 (8)
N1	0.0432 (9)	0.0451 (10)	0.0531 (9)	0.0044 (7)	0.0256 (7)	0.0105 (7)
C5	0.0418 (11)	0.0429 (11)	0.0363 (9)	−0.0046 (9)	0.0087 (8)	−0.0011 (8)
N3	0.0432 (10)	0.0605 (13)	0.0751 (12)	−0.0041 (9)	0.0245 (9)	−0.0147 (10)
C10	0.0500 (12)	0.0425 (12)	0.0400 (10)	−0.0027 (9)	0.0140 (9)	0.0011 (9)
C3	0.0407 (11)	0.0577 (13)	0.0467 (10)	−0.0116 (9)	0.0185 (8)	−0.0046 (9)
C2	0.0330 (9)	0.0492 (12)	0.0447 (10)	−0.0038 (8)	0.0162 (8)	−0.0006 (9)
C6	0.0633 (14)	0.0489 (12)	0.0395 (10)	0.0057 (10)	0.0116 (9)	0.0042 (9)
C1	0.0484 (11)	0.0557 (13)	0.0401 (10)	0.0045 (10)	0.0131 (8)	−0.0021 (9)
C9	0.0450 (11)	0.0535 (13)	0.0546 (12)	−0.0094 (10)	0.0183 (9)	−0.0029 (10)
N2	0.0551 (11)	0.0572 (13)	0.0725 (13)	−0.0050 (9)	0.0333 (10)	0.0088 (10)
O4	0.0646 (11)	0.0716 (12)	0.0967 (13)	0.0169 (9)	0.0250 (9)	−0.0032 (10)
C7	0.0537 (14)	0.0751 (17)	0.0443 (11)	0.0211 (12)	0.0041 (10)	−0.0059 (11)
O3	0.0672 (11)	0.0869 (14)	0.173 (2)	−0.0139 (10)	0.0806 (13)	−0.0085 (13)
C8	0.0367 (11)	0.0786 (17)	0.0633 (13)	−0.0060 (11)	0.0147 (10)	−0.0108 (12)
O1	0.0896 (13)	0.0543 (11)	0.1241 (16)	−0.0227 (9)	0.0458 (11)	−0.0050 (10)
O2	0.1054 (14)	0.1075 (15)	0.0614 (11)	0.0001 (11)	0.0347 (10)	0.0311 (10)

### Geometric parameters (Å, °)

**Table:**

O7—C10	1.251 (2)	C3—H3B	0.9700
O6—C10	1.261 (2)	C3—H3A	0.9700
O5—C5	1.351 (2)	C2—N2	1.496 (3)
O5—H5	0.8200	C2—C1	1.518 (3)
C4—C9	1.384 (3)	C6—C7	1.388 (3)
C4—C5	1.396 (2)	C6—H6	0.9300
C4—C10	1.503 (3)	C1—H1A	0.9700
N1—C1	1.493 (2)	C1—H1B	0.9700
N1—C3	1.495 (2)	C9—C8	1.372 (3)
N1—H1D	0.9000	C9—H9	0.9300
N1—H1C	0.9000	N2—O1	1.208 (2)
C5—C6	1.389 (3)	N2—O2	1.219 (2)
N3—O3	1.196 (2)	C7—C8	1.367 (3)
N3—O4	1.212 (2)	C7—H7	0.9300
N3—C2	1.517 (2)	C8—H8	0.9300
C3—C2	1.518 (3)		
			
C5—O5—H5	109.5	N2—C2—C1	116.46 (16)
C9—C4—C5	118.95 (17)	N3—C2—C1	114.04 (15)
C9—C4—C10	121.59 (17)	N2—C2—C3	116.25 (16)
C5—C4—C10	119.37 (16)	N3—C2—C3	114.66 (16)
C1—N1—C3	91.18 (13)	C1—C2—C3	89.36 (14)
C1—N1—H1D	113.4	C7—C6—C5	119.3 (2)
C3—N1—H1D	113.4	C7—C6—H6	120.3
C1—N1—H1C	113.4	C5—C6—H6	120.3
C3—N1—H1C	113.4	N1—C1—C2	89.66 (13)
H1D—N1—H1C	110.7	N1—C1—H1A	113.7
O5—C5—C6	118.36 (17)	C2—C1—H1A	113.7
O5—C5—C4	121.62 (16)	N1—C1—H1B	113.7
C6—C5—C4	120.02 (17)	C2—C1—H1B	113.7
O3—N3—O4	125.8 (2)	H1A—C1—H1B	110.9
O3—N3—C2	119.7 (2)	C8—C9—C4	121.03 (19)
O4—N3—C2	114.47 (17)	C8—C9—H9	119.5
O7—C10—O6	122.29 (18)	C4—C9—H9	119.5
O7—C10—C4	119.66 (17)	O1—N2—O2	126.9 (2)
O6—C10—C4	118.00 (17)	O1—N2—C2	116.75 (19)
N1—C3—C2	89.56 (14)	O2—N2—C2	116.36 (19)
N1—C3—H3B	113.7	C8—C7—C6	120.8 (2)
C2—C3—H3B	113.7	C8—C7—H7	119.6
N1—C3—H3A	113.7	C6—C7—H7	119.6
C2—C3—H3A	113.7	C7—C8—C9	119.87 (19)
H3B—C3—H3A	111.0	C7—C8—H8	120.1
N2—C2—N3	105.92 (15)	C9—C8—H8	120.1
			
C9—C4—C5—O5	178.87 (17)	O5—C5—C6—C7	−177.77 (16)
C10—C4—C5—O5	2.3 (2)	C4—C5—C6—C7	1.8 (3)
C9—C4—C5—C6	−0.7 (3)	C3—N1—C1—C2	−3.71 (15)
C10—C4—C5—C6	−177.31 (16)	N2—C2—C1—N1	−115.59 (16)
C9—C4—C10—O7	7.2 (3)	N3—C2—C1—N1	120.51 (16)
C5—C4—C10—O7	−176.32 (16)	C3—C2—C1—N1	3.65 (14)
C9—C4—C10—O6	−170.31 (17)	C5—C4—C9—C8	−0.3 (3)
C5—C4—C10—O6	6.2 (2)	C10—C4—C9—C8	176.18 (17)
C1—N1—C3—C2	3.71 (14)	N3—C2—N2—O1	87.1 (2)
O3—N3—C2—N2	−1.8 (2)	C1—C2—N2—O1	−40.8 (2)
O4—N3—C2—N2	178.59 (17)	C3—C2—N2—O1	−144.22 (18)
O3—N3—C2—C1	127.6 (2)	N3—C2—N2—O2	−91.5 (2)
O4—N3—C2—C1	−52.0 (2)	C1—C2—N2—O2	140.53 (18)
O3—N3—C2—C3	−131.4 (2)	C3—C2—N2—O2	37.1 (2)
O4—N3—C2—C3	49.0 (2)	C5—C6—C7—C8	−1.9 (3)
N1—C3—C2—N2	115.79 (17)	C6—C7—C8—C9	0.9 (3)
N1—C3—C2—N3	−119.95 (17)	C4—C9—C8—C7	0.2 (3)
N1—C3—C2—C1	−3.65 (14)		

### Hydrogen-bond geometry (Å, °)

**Table:**

*D*—H···*A*	*D*—H	H···*A*	*D*···*A*	*D*—H···*A*
N1—H1C···O6	0.90	2.38	2.922 (2)	118
N1—H1C···O7	0.90	1.81	2.708 (2)	179
N1—H1D···O7^i^	0.90	1.96	2.720 (2)	141

Symmetry codes: (i) −*x*+1, −*y*+2, −*z*.

## References

[bb1] Archibald, T. G., Gilardi, R., Baum, K. & George, C. (1990). *J. Org. Chem.* **55**, 2920–2924.

[bb2] Bruker (2003). *SMART* and *SAINT* Bruker AXS Inc., Madison, Wisconsin, USA.

[bb3] Gao, R., Ma, H. X., Yan, B., Song, J. R. & Wang, Y. H. (2009). *Chem. J. Chin. Univ.* **30**, 577–582.

[bb4] Hiskey, M. A., Coburn, M. D., Mitchell, M. A. & Benicewicz, B. C. (1992). *J. Heterocycl. Chem.* **29**, 1855–1856.

[bb5] Ma, H. X., Yan, B., Li, Z. N., Guan, Y. L., Song, J. R., Xu, K. Z. & Hu, R. Z. (2009). *J. Hazard. Mater.* **169**, 1068–1073. 10.1016/j.jhazmat.2009.04.057 19446396

[bb6] Ma, H. X., Yan, B., Li, J. F., Ren, Y. H., Chen, Y. S., Zhao, F. Q., Song, J. R. & Hu, R. Z. (2010). *J. Mol. Struct.* **981**, 103–110.

[bb7] Ma, H. X., Yan, B., Li, Z. N., Song, J. R. & Hu, R. Z. (2009). *J. Therm. Anal. Calorim.* **95**, 437–444.

[bb8] Ma, H. X., Yan, B., Song, J. R., Lü, X. Q. & Wang, L. J. (2009). *Chem. J. Chin. Univ.* **30**, 371–381.

[bb9] Sheldrick, G. M. (2000). *SADABS* . University of Göttingen, Germany.

[bb10] Sheldrick, G. M. (2008). *Acta Cryst.* A**64**, 112–122. 10.1107/S0108767307043930 18156677

[bb11] Yan, B., Ma, H.-X., Hu, Y., Guan, Y.-L. & Song, J.-R. (2009). *Acta Cryst.* E**65**, o3215. 10.1107/S1600536809049861 PMC2971829 21578923

[bb12] Yan, B., Ma, H.-X., Li, J.-F., Guan, Y.-L. & Song, J.-R. (2010). *Acta Cryst.* E**66**, o57. 10.1107/S1600536809051290 PMC2980018 21580159

